# Therapeutic Use of *Scoparia dulcis* Reduces the Progression of Experimental Osteoarthritis

**DOI:** 10.3390/molecules24193474

**Published:** 2019-09-25

**Authors:** Marcus Vinícius Viégas Lima, Abner de Oliveira Freire, Emerson Lucas Frazão Sousa, André Alvares Marques Vale, Alberto Jorge Oliveira Lopes, Cleydlenne Costa Vasconcelos, Mônica Virginia Viégas Lima-Aragão, Humberto Oliveira Serra, Rosane Nassar Meireles Guerra Liberio, Ana Paula Silva de Azevedo dos Santos, Gyl Eanes Barros Silva, Claúdia Quintino da Rocha, Fernando César Vilhena Moreira Lima, Maria do Socorro de Sousa Cartágenes, João Batista Santos Garcia

**Affiliations:** 1 Centro de Ciências Biológicas e da Saúde, Universidade Federal do Maranhão, São Luís 65080-805, Brazil; abnerfreire@live.com (A.d.O.F.); e.lucasfrazao@gmail.com (E.L.F.S.); andre_amvale@hotmail.com (A.A.M.V.); lopesajo@gmail.com (A.J.O.L.); cleydlenne@yahoo.com.br (C.C.V.); roguerra@globo.com (R.N.M.G.L.); apsazevedo@yahoo.com.br (A.P.S.d.A.d.S.); gyleanes@ig.com.br (G.E.B.S.); 2 Universidade Ceuma, São Luís 65075-120, Brazil; 3 Universidade Federal do Maranhão, Coordenação de Ciências Naturais, Campus Bacabal, São Luís 65080-80, Brazil; 4 Hospital Universitário Presidente Dutra, São Luís 65020-070, Brazil; hoserra@gmail.com; 5 Departamento de Química, Universidade Federal do Maranhão, São Luís 65080-805, Brazil; claudiarocha3@yahoo.com.br; 6 Faculdade Santa Terezinha, São Luís 65045-180, Brazil; fernandovilhena15@gmail.com

**Keywords:** *Scoparia dulcis*, Osteoarthritis, Pain, Inflammation, Treatment

## Abstract

Pain is recognized as one of the main symptoms in knee osteoarthritis and is the main reason why patients seek medical attention. *Scoparia dulcis* has been popularly used to relieve discomfort caused by various painful conditions. The objective of the study is to evaluate the analgesic and anti-inflammatory effect of the crude extract of *S. dulcis*, in an experimental model of osteoarthritis. The experiment was performed with Wistar rats divided into 4 groups with 5 animals each: healthy, saline, crude extract, and meloxicam groups. Knee osteoarthritis was induced by intra-articular injection of sodium mono-iodoacetate. First, clinical parameters of pain were assessed at days 0, 5, 10, 15, and 20 after induction. Second, the potential cyclooxygenase inhibition was evaluated, and the cytokines of the synovial fluid were quantified. An in silico test and Molecular Docking tests were performed. A histopathological evaluation was made on articular cartilage with safranin O staining. The results showed that a 15-day treatment with crude extract reduced edema, spontaneous pain, peripheral nociceptive activity, and proinflammatory cytokines in the synovial fluid. The highest inhibition of cyclooxygenase 2 in the crude extract occurred at 50 µg/mL. The crude extract of *S. dulcis* presents therapeutic potential for the treatment of osteoarthritis due to its anti-inflammatory and anti-nociceptive action.

## 1. Introduction

Osteoarthritis (OA) is a chronic, complex disease characterized by loss, alteration and progressive degeneration of cartilage and subchondral bone; reduction of joint space; synovitis; pain, and formation of osteophytes [1,2]. OA mainly affects the joints that are given greater weight, such as the knees, [3] It alters an individual’s the quality of life [4,5], is the most frequent cause of musculoskeletal disease and pain [6,7], and impacts daily living and work activities [8].

Although OA has long been defined as a degenerative disease characterized by increased pressure on a particular joint, the current understanding of OA has shifted from cartilage “wear and tear” to an inflammatory joint disease. Proinflammatory cytokines and chemokines have been shown to disrupt homeostasis in the cartilage matrix of OA patients, with increased production of interleukin-1 (IL-1)β and tumor necrosis factor (TNF)α by articular chondrocytes characteristic of established OA. In addition, IL-1β has been shown to induce chondrocytes to produce other inflammatory mediators, including IL-6 and nitric oxide, further amplifying detrimental cellular responses [9].

Advances in understanding the pathophysiology of OA, through the influence of biochemical stress, abnormal biomechanics of the joint, and the inflammatory pathways involved, have allowed an increase in therapeutic alternatives [10]. There has been a constant search for substances that can be combined with conventional therapy for OA. Currently, conventional therapy consists of a combination of nonpharmacological measures such as aerobic exercises, weight loss, and joint protection techniques, as well as symptomatic pharmacological treatments including anti- inflammatory nonsteroidal analgesics and corticosteroids or local intra-articular lubricants until, eventually, surgical intervention is required [6].

Medicinal plants and their derivatives represent a frequent alternative for the treatment of diseases [11]. Among these species, we highlight *Scoparia dulcis* (*S. dulcis*), also known as “*vassourinha*”, a perennial herb that is found in tropical and subtropical regions [12,13]. Its isolated bioactive constituents have contributed to demonstrate the plant′s medicinal effect by the presence of flavonoids, terpenes, tannins, saponins and steroids in inflammatory and nociceptive processes [14,15,16], which are the most widely used in the literature. *S. dulcis* has been used to relieve discomfort caused by menstruation, menopause [16], labor pain, uterine inflammation [17], and gastric lesions [18], although the exact mechanism of action remains unclear (15).

No published data on the anti-nociceptive and anti-inflammatory action of the plant species in treating pain caused by knee OA in rats have been found so far. Therefore, the objective of this research is to evaluate the analgesic and anti-inflammatory effect of the crude extract of *S. dulcis*, in an experimental model of OA.

## 2. Results

Twenty animals were divided into four experimental groups, five animals each: healthy group (GS): untreated healthy animals not induced for OA; saline group (GSAL): OA animals treated orally at 1 mL/kg/day with 0.9% sodium chloride (NaCl); *Scoparia dulcis* Group (GSD): OA animals treated orally with 500 mg/kg/day of crude *S. dulcis* extract; meloxicam group (GM): OA animals orally treated with 1 mg/kg/day meloxicam reported in the results below

### 2.1. Evaluation of Mechanical Allodynia—Von Frey

**Figure 1:**
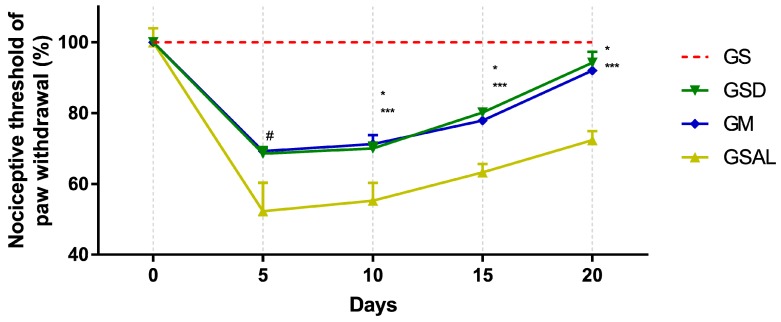
Effect of crude extract of *S. dulcis* on the tactile sensory evaluation using the Von Frey test. Results were expressed as mean ± standard mean error; # difference between healthy group (GS) and saline group (GSAL) in D5 (*p* < 0.0001), GS and *Scoparia dulcis* Group (GSD) in D5 (*p* < 0.0001), GS and meloxicam group (GM) in D5 (*p* < 0.0001); * difference between GSD and GSAL in D10 (*p* = 0.0015), D15 (*p* = 0.0002) and D20 (*p* < 0.0001); *** difference between GM and GSAL in D10 (*p* = 0.0004), D15 (*p* = 0.0016) and D20 (*p* < 0.0001), using 2-way ANOVA with the multiple comparisons test (*p* < 0.05).

Induction of OA reduced the nociceptive threshold of paw withdrawal in all groups in a similar way. However, after 10 days, nociceptive threshold increase and decrease of mechanical allodynia occurred in the GSD and GM groups, which presented values similar to GS at the end of 20 days (Figure 1).

### 2.2. Assessment of Motor Activity/Forced Walking—Rotarod test

**Figure 2:**
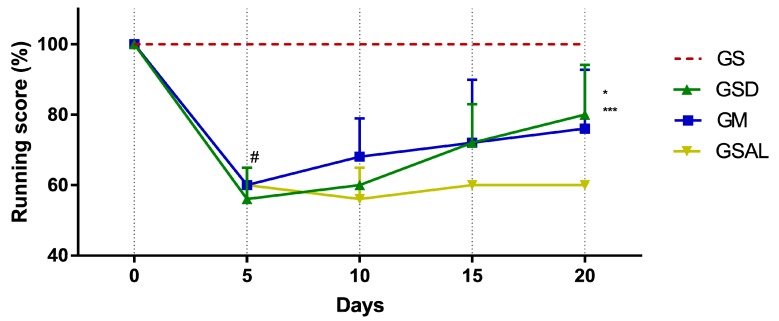
Effect of crude extract of *S. dulcis* on the evaluation of motor activity/forced ambulation using the Rotarod test. Results were expressed as mean ± standard mean error; # difference between GS and GSAL in D5 (*p* < 0.0001), GS and GSD in D5 (*p* < 0.0001), GS and GM in D5 (*p* < 0.0001); * difference between the GSD and GSAL in the D20 (*p* = 0.0061); *** difference between GM and GSAL in the D20 (*p* < 0.0472), using 2-way ANOVA with the multiple comparisons test (*p* < 0.05).

The induction of OA reduced gait score in all groups in a similar way. However, only on the 20th day did GSD and GM show an increase in their score (Figure 2).

### 2.3. Functional Incapacity—Weight Bearing Test

**Figure 3:**
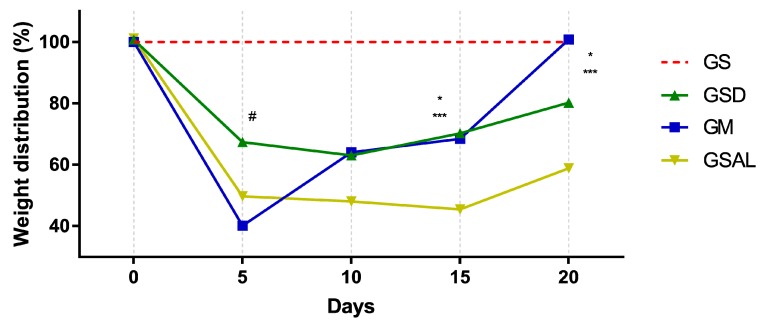
Effect of the crude extract of *S. dulcis* on the assessment of the weight distribution in the hind paws using the weight bearing test. Results were expressed as mean ± standard mean error; # difference between GS and GSAL in D5 (*p* < 0.0001), GS and GSD in D5 (*p* < 0.0001) and GS and GM in D5 (*p* < 0.0001); * difference between GSD and GSAL in D15 (*p* = 0.0025) and D20 (*p* = 0.0037); *** difference between GM and GSAL in D15 (*p* = 0.0062) and D20 (*p* < 0.0001), using 2-way ANOVA with the multiple comparisons test (*p* < 0.05).

Induction of OA reduced the nociceptive threshold of paw withdrawal in all groups in a similar way. However, after 15 days there was an increase in functional capacity (GSD and GM). At the end of 20 days GM is equal to GS (Figure 3).

### 2.4. Edematogenic Evaluation

**Figure 4:**
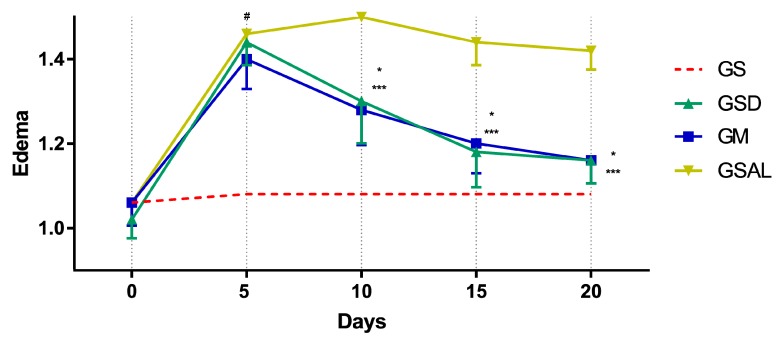
Effect of the crude extract of *S. dulcis* on the edematogenic evaluation of the circumference of the knee by the pachymeter. Results were expressed as mean ± standard mean error; # difference between GS and GSAL in D5 (*p* < 0.0001), GS and GSD in D5 (*p* < 0.0001) and GS and GM in D5 (*p* < 0.0001); * difference between GSD and GSAL in D10 (*p* < 0.0001), D15 (*p* < 0.0001) and D20 (*p* < 0.0001); *** difference between GM and GSAL in D10 (*p* < 0.0001), D15 (*p* < 0.0001) and D20 (*p* < 0.0001), using 2-way ANOVA with the multiple comparisons test (*p* < 0.05).

The induction of OA increased the circumference of the right knee characterizing edema in all groups in a similar way. However, after 10 days the edema reduction occurred in the treated groups (GSD and GM), which presented values similar to GS at the end of 20 days (Figure 4).

### 2.5. Rating by Mouse Grimace Scale

**Figure 5:**
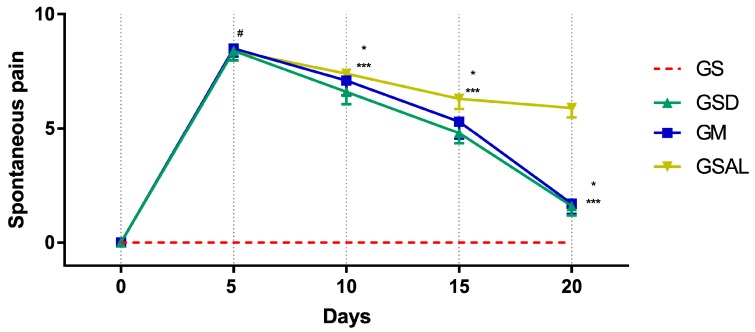
Effect of crude extract of *S. dulcis* on the evaluation of spontaneous pain by Mouse Grimace Scale. Results were expressed as mean ± standard mean error; # difference between GS and GSAL in D5 (*p* < 0.0001), GS and GSD in D5 (*p* < 0.0001), GS and GM in D5 (*p* < 0.0001); * difference between GSD and GSAL in D10 (*p* = 0.0062), D15 (*p* < 0.0001) and D20 (*p* < 0.0001); *** difference between GM and GSAL in D10 (*p* = 0.0068), D15 (*p* = 0.0003) and D20 (*p* < 0.0001), using 2-way ANOVA with the multiple comparisons test (*p* < 0.05).

The induction of OA increased spontaneous pain in all groups of the same magnitude. However, after 10 days the scores decreased in the treated groups (GSD and GM) (Figure 5).

### 2.6. Determination of Inhibition of Cyclooxygenase

**Figure 6:**
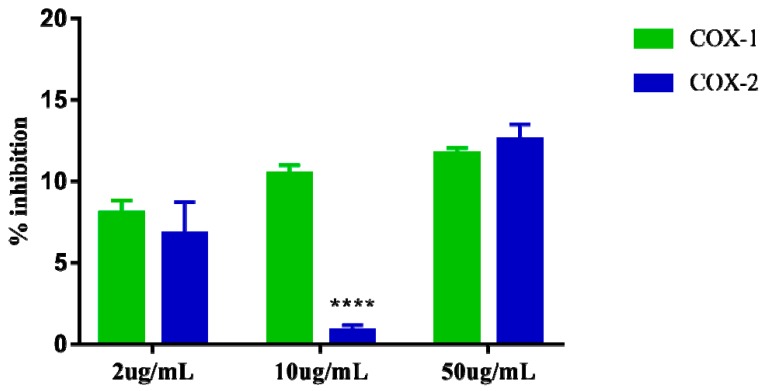
Percent inhibition of in vitro Cyclooxygenase-1 (COX-1) e Cyclooxygenase-2 (COX-2), induced by the crude extract of *S. dulcis*, tested in three concentrations: 2 μg/mL, 10 μg/mL, and 50 μg/mL. The data are represented in mean + standard deviation of the means. **** Represents significant differences, with *p* < 0.0001 comparing inhibition of COX-1 and COX-2.

Cyclooxygenase inhibition assays 1 and 2 showed that the extract at the highest concentration tested (50 µg/mL) can inhibit COX-1 by up to 11.69% and COX-2 by 12.57% suggesting that the extract apparently does not exert greater selectivity for one of these enzymes specifically. The results of the three concentrations (2, 10, and 50 µg/mL) evaluated for COX-1 inhibition also suggest that the extract appears to exert dose dependent effect. However, the results observed at the same concentrations (2, 10, and 50 µg/mL) for COX-2 inhibition do not appear to demonstrate the same dose-dependent effect, as the intermediate concentration 10 µg/mL showed very low inhibition. 0.83% for COX-2, which is lower than that observed for the dose of 2 µg/mL (Figure 6).

### 2.7. Chemical Analysis

**Figure 7:**
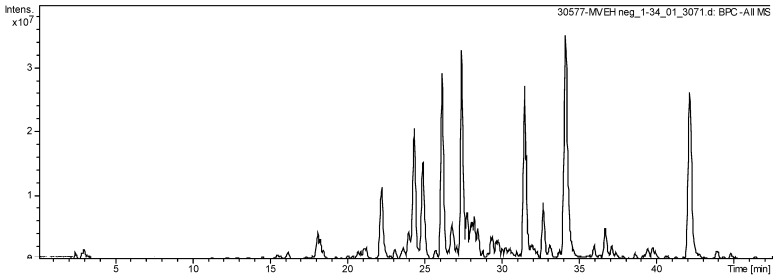
Total Ion Chromatogram of extract from *S. dulcis* acquired by LC-ESI-MS (*m*/*z*: 100–1500 Da).

The HPLC-ESI-MS analysis exhibit 18 secondary metabolites in the *S. dulcis* crude extract (Figure 7), highlighting flavonoids kaempferol derivates. Sixteen molecules were elucidated compounds (Table 1).

### 2.8. In Silico Assay

To our molecular docking analysis, we used all compounds identified by HPLC-MS on *S. dulcis* crude extract. In general, all compounds showed high parameter affinity with COX-2 structure, highlighting Suspensaside (−9.15 kcal.mol^−1^ and 0.19 µM) and Nicotiflorin (−8.26 kcal.mol^−1^ and 0.88 µM from binding energy and inhibition constant, respectively). In addition to these molecules, the molecular docking of the commercial non-steroidal anti-inflammatory drug meloxicam was performed, with affinity parameters of −8.89 kcal.mol^−1^ and 0.30 µM. The results of the binding energy values of all compounds are presented on Table 2.

**Figure 8:**
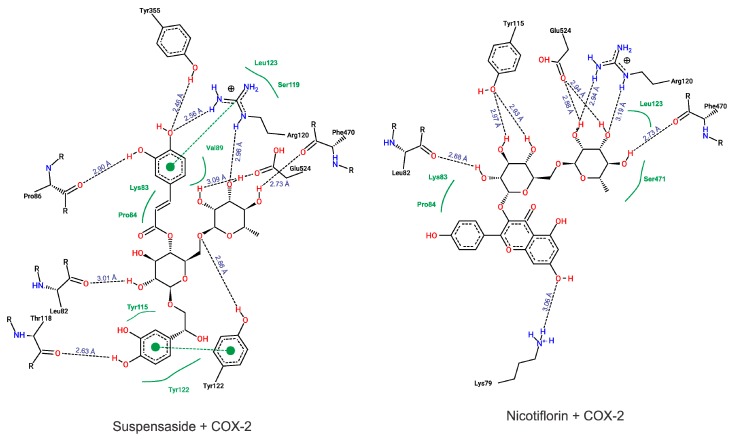
Diagram from interactions performed by Suspensaside and Nicotiflorin with COX-2 structure. Black dashed lines represent hydrogens bonds; green dashed lines represent pi-pi interactions and green full lines represent van der Walls interactions. The Figure was generated with PoseView.

**Figure 9:**
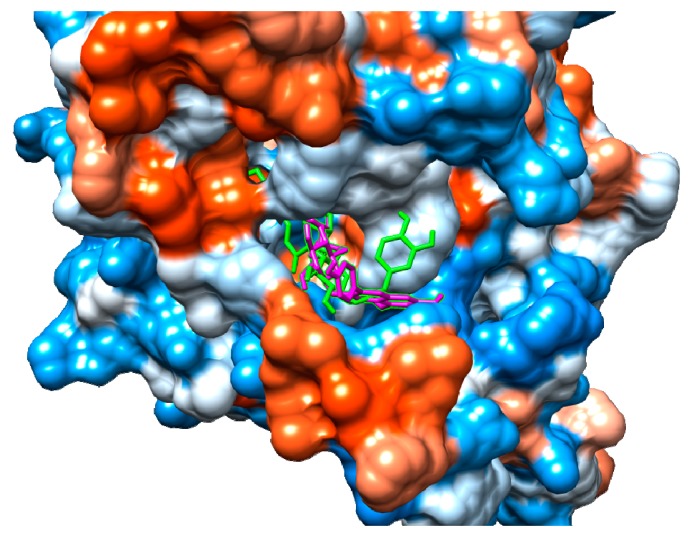
Spatial conformation of suspensaside (green) and nicotiflorin (magenta) on the COX-2 active site (PDB 1DDX). Image generated with USCF Chimera.

According to our results, both suspensaside and nicotiflorin performed interactions with important residues from COX active sites like Arg120 and Glu524 and with neighboring residues. Suspensaside performed more interactions of hydrogens bonds, and also performed pi-pi interactions. These interactions are energetically important. Suspensaside also performs seven van der Walls interactions while nicotiflorin, just 4 interactions (Figure 8). The spatial conformation of suspensaside and nicotiflorin with the COX-2 structure was shown on Figure 9.

### 2.9. Determination of Cytokines (in Synovial Fluid)

The animals treated with the crude extract and meloxicam showed a reduction of the cytokines IFN-γ and IL-6 when compared to the saline group that was statistically significant. As for interleukin 10, the crude extract and meloxicam groups presented a statistically significant increase when compared to the saline group (Table 3).

### 2.10. Microscopic Classification Articular Cartilage Histology in Osteoarthritis

**Figure 10:**
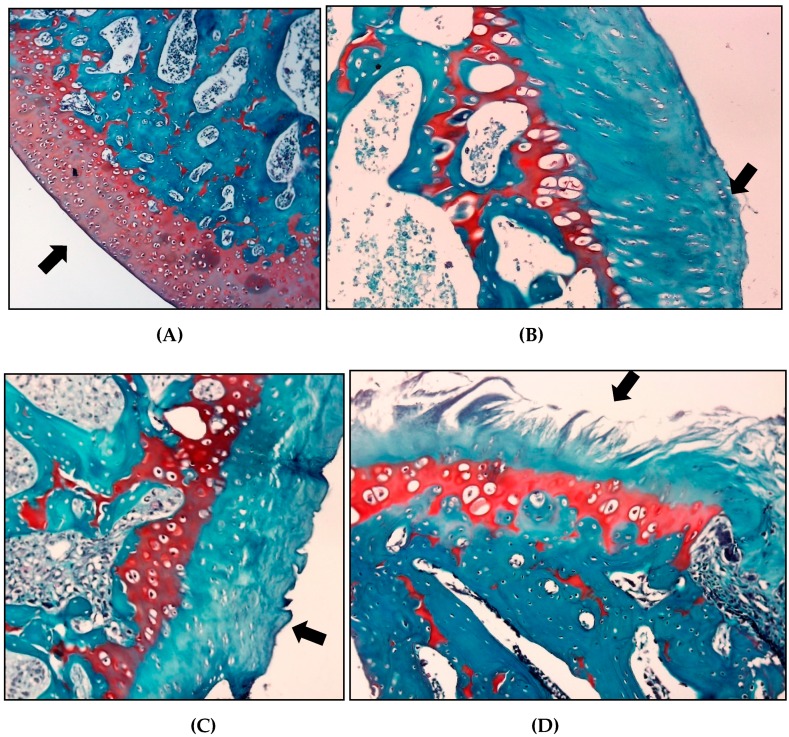
Effect of crude extract of *S. dulcis* on classification articular cartilage histology. (**A**) For articular cartilage, the score 0 represents the surface and morphology of intact cartilage (GS). (**B**) Score 1, an intact surface with superficial fibrillation (GSD). (**C**) For score 2 there is already a discontinuity of the articular cartilage (GM) surface. (**D**) For score 4 (GSAL), the surface has vertical crack-like cracks with erosion areas.

The histopathological evaluation using the OARSI scoring system for safranin *O*, the saline group showed a higher classification than the other groups (3.2 ± 0.95), indicating a greater involvement of the articular cartilage in these animals. The group treated with crude extract of *S. dulcis* and meloxicam presented mean scores of 2 ± 1.25 and 2.6 ± 0.81, respectively (Table 4 and Figure 10).

## 3. Discussion

This study aimed to evaluate the anti-nociceptive and anti-inflammatory effect of the crude extract of *S. dulcis* in an experimental model of knee OA in rats, with meloxicam as the positive control. It is the first study to evaluates the effect of crude extract of *S. dulcis* on pain and inflammation in knee OA in rats.

Throughout the study, the following markers were observed: an improvement in the gait score; the reduction of mechanical allodynia; the distribution of the weight discharge in the hind paws; signs of spontaneous pain and edema; reduction in inflammation; an increase in the peripheral nociceptive threshold; decrease in proinflammatory cytokines, inflammation and edema by synovial membrane histology; as well as a discontinuity of articular cartilage degradation.

The results of the present study showed similarity in the effects of the crude extract of *S. dulcis* to those of meloxicam, perhaps because they act more effectively in inhibiting the COX-2 and arachidonic acid cycle, which could result in decreased pain, inflammation, and degeneration of the articular cartilage by reducing primary nociception, edema, and the production of pro-inflammatory molecules [31].

According to Zulfiker et al. [32] all of the previously mentioned secondary metabolites isolated from the plant extract have a mechanism of peripheral action similar to non-steroidal anti- inflammatory agents such as indomethacin and diclofenac sodium. However, some compounds such as scoparic acid A, scoparic acid D, scutellarein, luteolin, coixol [33], scoparinol [34], glutinol [35], and betulinic acid [36] common in the plant species *S. dulcis* were not present in the crude extract.

Computational methods have been used as a quick and inexpensive alternative to experimentally screen large compound libraries to allow the identification of new target proteins from natural therapeutic products and thus reduce the number of experiments needed to determine their molecular mechanisms of action [37,38].

The anti-inflammatory effect of crude extract of *S. dulcis* through the suspensaside constituent appears to be closely related to inhibition of proinflammatory mediators (TNF-α, IL-1β, and IL-6), nitric oxide, and PGE_2_ in stimulated in lipopolysaccharide stimulated BV2 microglia cells through the activation of the Nrf2/HO-1 signaling pathway and down-regulation of NF-κB, JAK-STAT and p38 MAPK signaling pathways [39]. Nicotiflorine has antioxidant, anti-inflammatory, and neuroprotective effects by reducing proinflammatory cytokines, including TNF-α, IFN-γ, IL-1, and IL-6, which may explain the anti-inflammatory effect of the extract. [40].

Structurally, the active site of COX-2 consists of a lipophilic channel whose entry is formed by Arg120, Tyr355, and Glu524 residues [41] and its activation causes arachidonic acid metabolism from interacting with COX Arg120, Tyr355, Tyr385, and Ser530 residues, leading to prostaglandin production [42]. The molecular docking results shows that the secondary metabolites identified on *S. dulcis* crude extract interact favorably and spontaneously with these residues and as neighboring residues that are also important for interaction with drugs such as ibuprofen [43], meloxicam, and isoxicam [42]. This finding suggests that this plant species may be considered as a potential source in the search for new therapeutic alternatives.

In patients with OA, chondrocytes, as well as synovial cells, produce increased levels of inflammatory cytokines, such as interleukin 1β and TNF-α, which in turn decrease collagen synthesis and increase catabolic mediators, such as metalloproteinases and other inflammatory substances such as interleukin 8, interleukin 6, prostaglandin E_2,_ and nitric oxide. Moreover, mechanical stress, both by static and dynamic compression, increases the production of nitric oxide by the chondrocytes [44].

The cytokine IFN-γ is associated with the activation of microglial cells and nerve sensitization. IL-6 is a cytokine that promotes maturation and activation of neutrophils and macrophages, as well as differentiation/maintenance of cytotoxic T lymphocytes and natural killer cells. It also activates astrocytes and microglia in the dorsal region of the spinal cord [45].

The decrease in IFN-γ and IL-6 cytokines in the present study suggests that there was a reduction in spinal cord neuroinflammation, which can lead to the attenuation of pain signals and improvement of hypersensitivity and hyperalgesia.

IL-10 is an anti-inflammatory cytokine that inhibits proinflammatory cytokines, mainly TNF, IL- 1, and IL-6, enhances the proliferation of mast cells, and prevents the production of IFN-γ by natural killer cells [46]. The increase in IL10 in synovial fluid may be related to decreased IL-6 and IFN-γ levels in the study and may be associated with improved clinical signs, with a possible reduction in inflammation and increase in peripheral circulation and nociceptive threshold.

Prostaglandins E_2_ and leukotrienes when released play a key role in the genesis of signs and symptoms of the inflammatory process, such as spontaneous edema and pain, which are evaluated in the crude extract group in the study, in addition to hypersensitizing the polymodal nociceptors of the C fibers to mechanical stimuli, chemical [47] and cytokines [48]. A possible inhibition of COX-2 by the *S. dulcis* extract seems to suggest a decrease in the production of prostaglandins E_2_ in the joint, showing its possible similarity to the positive control of the study.

Decreasing IL-6 and IFN-γ cytokines may have led to a progressive improvement in mechanical allodynia over the 15 days of the study, but other unstudied mechanisms may be associated with pain.

Functional incapacity and forced motor activity/ambulation improved only between the 15th and 20th day of treatment. The proliferation of fibrous tissue in the knee, as demonstrated by the synovial membrane histology of the present study, can cause biomechanical and functional alterations that can result in a postural adjustment to compensate and protect the injured knee by, reducing the weight to maintain the joint in flexion and, thus minimizing pain [49].

Another possibility for this result includes the decrease in joint space caused by sodium mono-iodoacetate induction (MIA), which could lead to joint misalignment and concentration of intra-articular stress, and thus, increase the impact of weight discharges on ambulation, increased friction in the joint articulation, which increases or maintains joint pain, and possibly a compensatory postural adjustment [50], as seen in the functional incapacity test.

During the inflammatory process, there is an increase in vascular permeability, capillary extravasation, and cell migration [51] as demonstrated by the histology of the study. Thus, a classic sign of the inflammatory process is the formation of edema that can continue for days and reduce joint mobility [52].

A histopathological analysis of the synovial membrane and articular cartilage showed encouraging results, suggesting the possible efficacy of *S. dulcis* in the treatment of OA at the dose tested. Hyaline cartilage is the most relevant articular tissue in the pathogenesis of this disease [6]. Although OA is characterized by subchondral bone sclerosis, it is uncertain that bone changes are the cause or consequence of the lesions, given that the cartilage properties depend on the bone bed, which can be affected by the mechanical function of the cartilage [53].

Velosa et al. [54] reported that up to now, OA treatment is based on drugs that control the pain and inflammation associated with synovitis, but do not reduce the destruction of the articular cartilage. Therefore, a herbal remedy with the potential to protect this tissue has excellent clinical relevance. Thus, the possible protective effect of the crude extract of *S. dulcis* on the cartilage demonstrated in the present study may justify the translation of its use for this purpose.

The reduction of the proinflammatory cytokines in synovial fluid may be associated with a reduction in the inflammatory infiltrate in the synovial membrane and, consequently, reduction of edema, as our results show, since synovial inflammation in OA is usually found next to pathologically damaged areas with bone and cartilage, releasing proteinases and cytokines capable of accelerating joint destruction [55].

Corroborating our results, Nagy et al. [56] in an MIA-induced mouse OA model reported that low doses (0.2 mg/kg) and high doses of meloxicam (1 mg/kg) had a chondroprotective effect and that high doses, also protected against subchondral bone lesions, suggesting the interruption of the low grade inflammatory pathway accompanied by chronic deterioration of cartilage, as shown by the possible effects of the crude extract in the study.

Changes in the subchondral bone and bone marrow support and perpetuate the deterioration of cartilage [56], thus the possible chondroprotective effect of *S. dulcis*, suggests that oral therapy with a dose of 500 mg/kg of crude extract may attenuate the progression of the disease in the chronic phase.

The effects of the crude extract of *S. dulcis* on histological progression, pain behavior, and inflammatory process in OA presented in the study are impressive, however, this work had some limitations that need to be highlighted, such as not evaluating the antioxidant activity of the crude extract by enzymatic and non-enzymatic methods. Also, in future studies, a centrally acting medication could be used as a positive control, in addition to evaluating different doses and their possible effects on the inhibition of COX-1 and COX-2 and possible toxic effects.

## 4. Materials and Methods 

The study was conducted at the Experimental Laboratory for Study of Pain (LEED), after approval by the Ethics Committee on Animal Use of the Federal University of Maranhão—Brazil (CEUA-UFMA number 23115.018030/2014-94).

### 4.1. Animals

Twenty male approximately 60-day-old Wistar rats, *Rattus norvegicus* species (albinus variety) were used in the study. The animals were obtained from the Central Animal Facility of the Universidade Federal do Maranhão. They remained in the vivarium of the LEED and were fed a standard ration and water ad libitum and maintained under controlled conditions of light and temperature.

### 4.2. Plant Species

The aerial parts of *S. dulcis* (2 kg) were collected at Cidade Universitária Dom Delgado at the Federal University of Maranhão (UFMA), city of São Luís (MA, USA) (Latitude: 02°31′47″ S Longitude: 44°18′10″ W and Altitude: 24 m) in April 2017 under similar conditions of climate and temperature [35,57]. The species was identified in the “Atico Seabra” herbarium at UFMA under exsiccate no. 7426.

### 4.3. Obtaining Extract from the Aerial Parts of Scoparia dulcis

The aerial parts of *S. dulcis* were oven dried with forced air circulation at 45 °C under constant weight and pulverized in a knife mill to obtain a moderately thick powder (666.66 g). The obtained powder which underwent an extraction process by maceration, was subjected to the drug/solvent ratio of 1:4 (*w*/*v*) with 2400 mL of ethanol for 48 h. The extractive solution was filtered and concentrated in a rotary evaporator under vacuum (40 °C) to obtain a 70% ethanol extract from the aerial parts of *S. dulcis* [35,57].

### 4.4. Experimental Protocol

Twenty animals were divided into four experimental groups with, 5 animals each: sadio group (GS): untreated and uninduced healthy animals for OA; salina group (GSAL): OA animals treated orally, with 1 mL/kg/day with 0.9% sodium chloride (NaCl); *Scoparia dulcis* group (GSD): animals with OA treated orally, with 500 mg/kg/day of crude extract of *S. dulcis*; meloxicam group (GM): animals with OA treated orally with 1 mg/kg/day of meloxicam.

### 4.5. Sodium MIA-Induced OA Model

For the induction of OA, the animals were anesthetized with intraperitoneal injections of 40 mg/kg of sodium thiopental. After certifying the anesthetic plane, a trichotomy was performed in the right knee and, subsequently, a topical solution of 10% iodopovidone was applied for local asepsis. An articular lesion was induced by a single intra-articular injection of 2 mg of sodium MIA (diluted in a maximum volume of 25 μL) into the right knee through the patellar ligament [58,59].

### 4.6. Clinical Evaluations

#### 4.6.1. Motor Activity Assessment—Forced Walking (Rotarod Test)

The animals were placed in a Rotarod (IITC Life Science, Woodland Hill, CA, USA) at a rate of 16 revolutions per minute for a period of 300 s. Use of the affected limb was assessed through forced walking. Use of the homolateral paw for MIA induction was graded by a subjective measure that employed a numerical scale ranging from 5 to 1 (5 = normal paw use; 4 = mild limping; 3 = severe limping; 2 = intermittent disuse of affected paw; 1 = complete disuse of affected paw) [60].

#### 4.6.2. Weight-Bearing Test/Weight Distribution Test on Hind Legs

The animals were placed in a glass bowl angled and positioned so that each hind leg lay on different platforms. The weight exerted on each back paw (measured in grams) was evaluated for 5 s. The final measurement of weight distribution was the mean of 3 measurements [58].

#### 4.6.3. Quantification of Mechanical Allodynia (Von Frey Test)

The evaluation of mechanical allodynia was performed using an electronic device (Model 1601C, Life Science, San Francisco, CA, USA), which consisted of a pressure transducer connected to a digital force counter expressed in grams (g) and calibrated to record a maximum force of 150 g. The animals were placed in individual transparent acrylic boxes on raised platforms to allow access to the lower part of their bodies. The holes in the platforms provided access to the transducer tip, allowing its contact with the animals′ paws. The frequency of the paw′s withdrawal response to the filament stimulus was measured in 5 applications, lasting 1s each, always performed by the same evaluator, and the final result was the mean of all measurements [60,61].

### 4.7. Mouse Grimace Scale (MGS)

The MGS a new method to evaluate spontaneous pain in animals in the laboratory through changes in facial expressions. To discriminate the subjective sensation of facial pain, the following evaluation criteria were adapted from Sotocinal et al. [62]: absent pain equals “0”; moderate pain equals “1”, and severe pain equals “2” through identification of eye changes, changes in nose/cheek protuberance, changes in ears and changes in mustache.

### 4.8. Edematogenic Evaluation

To evaluate the anti-edematogenic effect, a Starrent^®^ brand caliper was used to quantify the knee joint diameter in millimeters. The pachymeter was positioned on the joint line to check the transverse diameter of the knee. The measurement was performed 3 times in each knee and the mean difference between the 2 limbs was used as the final result [63].

### 4.9. Cyclooxygenase (COX) Inhibition

The assay was performed according to the manufacturer′s recommendations (Cox colorimetric inhibitor screening—Cayman chemical^®^) in 96-well plates. To determine the percentage of inhibition of the extract, which was ascertained from the absorbance data and obtained by reading the plate at 590 nm, initially the BW mean value was subtracted from the absorbance means of A (A-BW) and absorbance of each extract at each concentration tested (Absorbance of extract sample [x]-BW). Then, the percentage of inhibition per se was determined by subtracting and dividing the value of each sample (crude extract of *S. dulcis*, at each concentration tested, subtracted the BW value) from the mean absorbance value of A (already subtracted BW), and multiplying by 100.

### 4.10. Chemical Analysis of Crude Extract of S. dulcis

The *S. dulcis* crude extract was analyzed by HPLC (LC-20AD Shimadzu, Kyoto, Japan) and a Phenomenex Luna C-18 (250 × 4.6 mm − 5 um) column. The mobile phases consisted of ultrapure water containing 0.1% formic acid (A) and methanol (B). The following linear gradient was applied: 0 min, 5% B; 1–60 min, 5–100% B; 60–70 min, 100% B at flow rate of 1 mL/min. The LC was coupled to a mass spectrometer (Amazon Speed ETD, Bruker, MA, USA) equipped with electrospray ionization (ESI) and an ion-trap (IT) type analyzer in negative mode, under the following conditions: capillary voltage at 4.5 kV, capillary temperature of 325 °C, entrainment gas (N2) flow at 12 L/min, and nitrogen nebulizer pressure at 27 psi. The acquisition range was *m*/*z* 100–1000, with 2 or more events.

### 4.11. In silico Assay

#### 4.11.1. Predictive Models and Theoretical Calculations

The compounds identified in the crude extract of *S. dulcis* had their geometric, electronic and vibrational properties optimized using the Gaussian program 09 [64]. The GaussView 5.0.8 [65] was used to obtain 3D structural models. Geometric optimization calculations were performed according to the functional density theory (DFT) method, which combine the functional hybrid B3LYP and the set of bases 6-31 ++ G (d, p).

#### 4.11.2. Molecular Docking

All docking procedures utilized Autodock 4.2 package [66,67]. The structure of the cyclooxygenase 2 (COX-2) (PDB ID 1DDX) and ligands were prepared for docking simulations with AutoDock Tools, version 1.5.6 [67]. Docking methodology described in the literature was used with modifications [11,59]. Gasteiger partial charges were calculated after adding all hydrogens. Non- polar hydrogens from COX-2 and *S. dulcis* compounds were subsequently merged. The dimensions of the cubic box in the X-, Y- and Z-axes were 120 Å × 120 Å × 120 Å, respectively, with a spacing of 0.375 Å between grid points. The grid box was centered on residue Arg120 from COX-2 and the Lamarckian genetic algorithm (LGA) was chosen to search for the best conformations, with 100 runs for each compound. Initial coordinates of COX-2 and *S. dulcis* compounds interactions were chosen using the criterion of lowest docking conformation of cluster with lowest energy combined with visual inspection.

### 4.12. Laboratory Analysis of Cytokines

A laboratory analysis of the synovial fluid to quantify IL-6, IL-10, TNF-α, and IFN-γ was performed using an enzyme-linked immunosorbent assay (ELISA, R&D Systems^®^, Minneapolis, MN, USA).

The synovial fluid samples were obtained on D28 by washing out the affected knee joint twice with 200 μL of phosphate-buffered solution (0.15 M, pH 7.4) containing 37.2 mg of ethylenediaminetetra-acetic acid (EDTA, 0.01 M).

### 4.13. Histopathological Analysis of Articular Cartilage

On day 21, the articular cartilage and subchondral bone of the knee of each animal were removed after euthanasia. The excised components were embedded in paraffin blocks and cut into 5 μm sections, and the proteoglycans of the organic cartilage matrix were specifically stained using 0.5% safranin-*O*.

The histopathological evaluation was performed according to the guidelines of the Osteoarthritis Research Society International (OARSI). The slides were analyzed blindly by two pathologists, who graded them on a scale of 0–6, according to the severity of the articular cartilage lesion. The classification considered the most severe lesion observed on the slide regardless of the extent of the lesion. Grade 0 indicated morphologically intact cartilage, grade 1 indicated an intact surface with possible focal lesions or abrasion, grade 2 showed discontinuity in the articular surface, grade 3 showed vertical fissures, grade 4 presented erosion, grade 5 exhibited denudation with sclerotic bone or fibrocartilaginous tissue repair or both, and grade 6 showed remodeling and bone deformation with changes in the contour of the articular surface [68,69].

### 4.14. Statistical Analysis

A comparison of the means of the experimental groups was performed with a univariate analysis of variance (One-way ANOVA), followed by the multiple comparisons test. In the evaluation of 2 sources of variability, a bivariate variance analysis (2-way ANOVA) was used. Data were analyzed using GraphPadInstal^®^ 7.0 software (GraphPad software, San Diego, CA, USA) and all analyses had a significance level of *p* < 0.05.

## 5. Conclusions

Oral treatment with the crude extract of *S. dulcis* reduced edema, spontaneous pain, and peripheral nociceptive activity in the right knee OA, attenuating the histological changes in the synovial membrane and the articular cartilage with lower synovial levels of IL-6 and IFN-γ, and higher levels of IL-10, possibly due to its anti-inflammatory action.

The tested concentration of 50 µg/mL showed the highest inhibition for COX-1 and COX-2 in the crude extract. All compounds showed high parameter affinity with the COX-2 structure, highlighting suspensaside and nicotiflorin and performed interactions with important residues from the COX active site, such as Arg120 and Glu524 and with neighboring residues.

In the challenging search for drugs that modify the pathogenesis and history of OA progression, preclinical results from the present study are encouraging and support the potential use of the crude extract of *S. dulcis* as a promising therapeutic agent that may complement or serve as an alternative to drug treatment. Further studies should be performed evaluating other doses, extracts, and fractions, as well as toxicity and other possible mechanisms of action.

## Figures and Tables

**Table 1:** Identification of compounds in *S. dulcis* crude extract by LC-ESI/MS.

Peak	Tr (min)	[M − H] ^−^	MS^n^ Fragments	Compound	Reference
1	18.0	417	285; 241; 152	kaempferol-3-*O*-pentoside	[19]
2	20.0	327	164; 136	gonzalitosin I	[20]
3	22.3	593	575; 473; 353	nicotiflorin	[21]
4	22.3	639	621; 529; 459	suspensaside	[22]
5	24.0	563	545; 473; 353	apigenin 6-C-pentosyl-8-C-hexoside	[23]
6	24.1	631	563, 479	myricetin-3-*O*-(2″-*O*-galloyl) glucoside	[24]
7	24.4	1127	563	acteoside dimer	[25]
8	26.2	623	563	acteoside (verbascoside)	[25]
9	26.2	623	461; 315	scoparin 7-*O*-glucoside	[26]
10	26.7	563	473; 443; 353	apigenin 6-C-pentosyl-8-C-hexoside isomer	[23]
11	27.4	769	623; 607; 461	deoxyrossicaside A	[27]
12	27.4	791	445; 283	biochanin A *O*-hexoside-*O*-hexoside	[23]
13	31.5	683	637; 313	Ginsenoside F1	[28]
14	31.5	751	705; 381	N.I*	
15	32.6	797	621; 475	N.I*	
16	36.7	299	284	5,7,4′-trihydroxy-3′-methoxyisoflavone (Rhamnocitrin)	[23]
17	42.1	313	297; 283	Diosmetin	[29]
18	44.1	327	291; 229; 211; 171	trihydroxyoctadecadienoic acid	[30]

* Not identified.

**Table 2:** Free-binding energies and inhibition constant obtained by molecular docking of the compounds identified in the *S. dulcis* crude extract with the COX-2 structure.

Ligand	ΔGbind (kcal.mol^−1^) *	Ki (μM) **
Suspensaside	−9.15	0.19
Nicotiflorin	−8.26	0.88
Diosmetin	−7.97	1.44
Rhamnocitrin	−7.72	1.81
Gonzalitosin I	−7.54	1.98
Kaempferol-3-*O*-pentoside	−7.45	3.44
Acteoside	−7.33	4.22
Meloxicam	−8.89	0.30

* ΔGbind, binding energy. ** Ki, inhibition constant.

**Table 3:** Effect of crude extract of *S. dulcis* on cytokines.

Cytokines	GSD	GM	GSAL	GS
**IFN-γ**	1387 ± 71.06 (*p* = 0.0090) *	1559 ± 117.9 (*p* = 0.3103)	1748 ± 195.7	1302 ± 157.8
**IL-6**	35.46 ± 6.78 (*p* = 0.0300) *	32.19 ± 11.54 (*p* = 0.0106) ***	55.91 ± 8.08	24.73 ± 9.92
**IL-10**	384 ± 28.09	390.5 ± 22.62	276.8 ± 46.76	373.2 ± 31.17
	(*p* = 0.0035) *	(*p* = 0.0019) ***		

* GSD and GSAL for IFN-γ (*p* = 0.0090), IL6 (*p* = 0.0300) and IL10 (*p* = 0.0035); ^***^ GM and GSAL for IL6 (*p* = 0.0106) and IL10 (*p* = 0.0019), using One-way ANOVA with the multiple comparisons test (*p* < 0.05).

**Table 4:** Effect of crude extract of *S. dulcis* on articular cartilage.

Variables	GSD	GM	GSAL	GS
**Degree**	2 ± 1.25	2.6 ± 0.81	3.2 ± 0.95	0 ± 0
***p*-value (GS)** ***p*-value (GSAL)**	(*p* = 0.0006) * (*p* = 0.0492)+	(*p* < 0.0001) *** (*p* = 0.5742)		

* Difference between GSD and GS for histology of articular cartilage (*p* = 0.0006); *** Difference between GM and GS for histology of articular cartilage (*p* < 0.0001). + Difference between GSD and GSAL for histology of articular cartilage (*p* = 0.0492), using One-way ANOVA with the multiple comparisons test (*p* < 0.05).
